# Human electrocortical, electromyographical, ocular, and kinematic data during perturbed walking and standing

**DOI:** 10.1016/j.dib.2021.107635

**Published:** 2021-11-25

**Authors:** Steven M. Peterson, Daniel P. Ferris

**Affiliations:** a Department of Biology, University of Washington, Seattle 98195, USA; b Department of Biomedical Engineering, University of Florida, J. Crayton Pruitt Family, Gainesville 32611, USA

**Keywords:** Mobile brain/body imaging, Electroencephalography, Electromyography, Electrooculography, Motion capture, Independent component analysis

## Abstract

Active balance control is critical for performing many of our everyday activities. Our nervous systems rely on multiple sensory inputs to inform cortical processing, leading to coordinated muscle actions that maintain balance. However, such cortical processing can be challenging to record during mobile balance tasks due to limitations in noninvasive neuroimaging and motion artifact contamination. Here, we present a synchronized, multi-modal dataset from 30 healthy, young human participants during standing and walking while undergoing brief sensorimotor perturbations. Our dataset includes 20 total hours of high-density electroencephalography (EEG) recorded from 128 scalp electrodes, along with surface electromyography (EMG) from 10 neck and leg electrodes, electrooculography (EOG) recorded from 3 electrodes, and 3D body position from 2 sensors. In addition, we include ∼18000 total balance perturbation events across participants. To facilitate data reuse, we share this dataset in the Brain Imaging Data Structure (BIDS) data standard and publicly release code that replicates our previous event-related findings.

## Specifications Table

**Table:** 

Subject	Neuroscience: General
Specific subject area	Human mobile brain/body imaging
Type of data	Multidimensional time-series recordings from electroencephalography (EEG), electromyography (EMG), electrooculography (EOG), and motion capture, along with balance perturbation onset timings, output matrices from independent component analysis, and relevant descriptive metadata
How data were acquired	BiomSemi ActiveTwo system with gelled electrodes, Biometrics Ltd wired surface EMG sensors, multi-camera Vicon motion capture system, Omega Engineering LCM703 load cells, Lab Streaming Layer, Unity 5 software
Data format	Raw Processed
Parameters for data collection	The treadmill-mounted balance beam was 2.5 cm tall and 12.7 cm wide. During walking sessions, we set the treadmill speed to 0.22 m/s. Two sensorimotor perturbations were used: a 0.5 s, 20° visual field rotation and a 1 s mediolateral pull of ∼15 Newtons. EEG, leg EMG, EOG, and motion capture recordings were originally sampled at 512 Hz, 1000 Hz, 512 Hz, and 100 Hz, respectively.
Description of data collection	Thirty participants performed four 10 min sessions of standing or walking on a treadmill-mounted balance beam while having their balance perturbed by either virtual-reality-induced visual field rotations or side-to-side waist pulls. Each session includes 150 rotation or pull perturbations (75 in each direction). EEG, EMG, EOG, and motion capture were collected (149 total channels) and synchronized using a 2 s square wave signal. Each data file contains raw and minimally processed data, along with identified noisy electrodes, independent component analyses weight matrices, and perturbation event onset times.
Data source location	University of Michigan Ann Arbor, Michigan United States
Data accessibility	All data files are publicly available on OpenNeuro under data identification number *ds003739* (https://openneuro.org/datasets/ds003739/) [1]. Our Matlab analysis scripts are freely available at http://doi.org/10.5281/zenodo.5701797[2].
Related research article	S.M. Peterson, D.P. Ferris, Differentiation in theta and beta electrocortical activity between visual and physical perturbations to walking and standing balance, eNeuro e0207-18.2018 (2018) 1-20. https://doi.org/10.1523/ENEURO.0207-18.2018

## Value of the Data

• Our dataset contains multiple human biosignals, including high-density EEG, that can be used to further our understanding of how the human body adapts to unexpected balance perturbations during a mobile beam-walking task.
• This multi-modal dataset can benefit researchers interested in the neural correlates of balance control, the physiological effects of virtual reality headsets, sensory integration, corticomuscular connectivity, mobile neuroimaging, and noninvasive neural decoding.
• Our data is formatted in the Brain Imaging Data Structure (BIDS) data standard to facilitate data reuse, and we provide freely accessible code to replicate the findings from our related research article.
• We include data from 30 participants, each with 149 data channels and ∼600 perturbation events, which can be used to benchmark signal processing techniques for mobile tasks and assess inter-participant variability across multiple recording modalities and tasks.
• We have precisely synchronized all recording modalities in this dataset, enabling researchers to explore how eye movements, head position, and neck muscle activity contribute to EEG motion artifact during mobile tasks.
• The multi-modal aspect of our dataset also provides an opportunity to explore sensor fusion and multi-modal decoding strategies for robust, noninvasive brain-computer interfaces.

## 1 Data Description

Our dataset contains high-density electroencephalography (EEG), electrooculography (EOG), neck/leg electromyography (EMG), and motion capture recordings from 30 human participants during sensorimotor balance perturbations. Participants either stood or walked on a treadmill-mounted balance beam while experiencing brief visual field rotation or side-to-side pull perturbations. For each participant, we recorded four 10 min sessions: (1) standing during pull perturbations (*pull stand*), (2) standing during rotation perturbations (*rotate stand*), (3) walking at 0.22 m/s during pull perturbations (*pull walk*), and (4) walking at 0.22 m/s during rotation perturbations (*rotate walk*) [3]. All data files are separated by session for each participant and formatted in the Brain Imaging Data Structure (BIDS) data standard to facilitate data reuse [4], [5].

All biosignal data recordings are saved as EEGLAB *.set* and *.fdt* files. The *.fdt* files contain the time-series data, while the *.set* files include relevant metadata. Both data files can be loaded into Matlab using EEGLAB [6]. Once loaded into EEGLAB, the *data* field will contain the time-series data. The first 128 rows of this field contain EEG data, ordered according to BioSemi’s 128-channel layout (https://www.biosemi.com/headcap.htm). The next rows are neck EMG (2 rows), EOG (3 rows), leg EMG (8 rows), three-dimensional position at the head and sacrum (6 rows), and finally pull force recordings (2 rows). The specific label, data type, and units for each row can be found in the *_channels.tsv* file in the same folder as the *.set* and *.fdt* files. In addition, the *_electrodes.tsv* file contains precisely measured three-dimensional positions for all EEG, neck EMG, and EOG electrodes in meters.

During each 10 min recording session, participants were exposed to 150 perturbation events. Only one type of perturbation (pull or rotation) occurred in each session. Event information can be found in the *event* field after opening the *.set*/*.fdt* files or in the *_events.tsv* file in the same folder. For each event, we provide the type of event, onset time, and duration. The type of event includes both type of perturbation performed (pull or rotation) and which direction of the perturbation (left or right for pulls, clockwise or counterclockwise for rotations).

We also include relevant noisy electrode and source localization information in the *etc* field after opening the *.set*/*.fdt* files. The *etc.good_chans* field contains the indices of all electrodes identified as not noisy, based on the criteria listed in the next section. We additionally provide the weight and sphering matrices from running adaptive mixture independent component analysis [7], which can be found in *etc.icaweights* and *etc.icasphere*, respectively. The *etc.good_comps* field includes the indices of the independent components that both authors agreed represent neural sources based on visual inspection of power spectra shape (decreasing power with increasing frequency [8]) and position within the head. Finally, we include the estimated DIPFIT2 equivalent dipole information for each independent component in the *etc.dipfit* field [9].

## 2 Experimental Design, Materials and Methods

We collected data from 30 healthy, young adults (15 females, 15 males; 22.5 ± 4.8 years old [mean ± SD]). All participants self-identified as right hand/foot dominant and had normal or corrected vision. We screened participants for any orthopedic, neurological, or cardiac conditions as well as for motion sickness in virtual reality. All participants provided written informed consent. Our protocol was approved by the University of Michigan Institutional Review Board.

### 2.1 Experimental design

Participants underwent four 10 min recording sessions of either standing or walking on a treadmill-mounted balance beam. The balance beam was 2.5 cm tall and 12.7 cm wide, which enforced tandem gait and tandem stance. In all sessions, participants wore a body-support harness for safety and crossed their arms. We instructed participants to move only their hips side-to-side while balancing and to avoid rotating across the longitudinal axis of their body [10], [11]. During walking sessions, participants walked heel-to-toe at 0.22 m/s. For standing sessions, we instructed participants to stand with their right foot in front of their left foot.

**Fig. 1:**
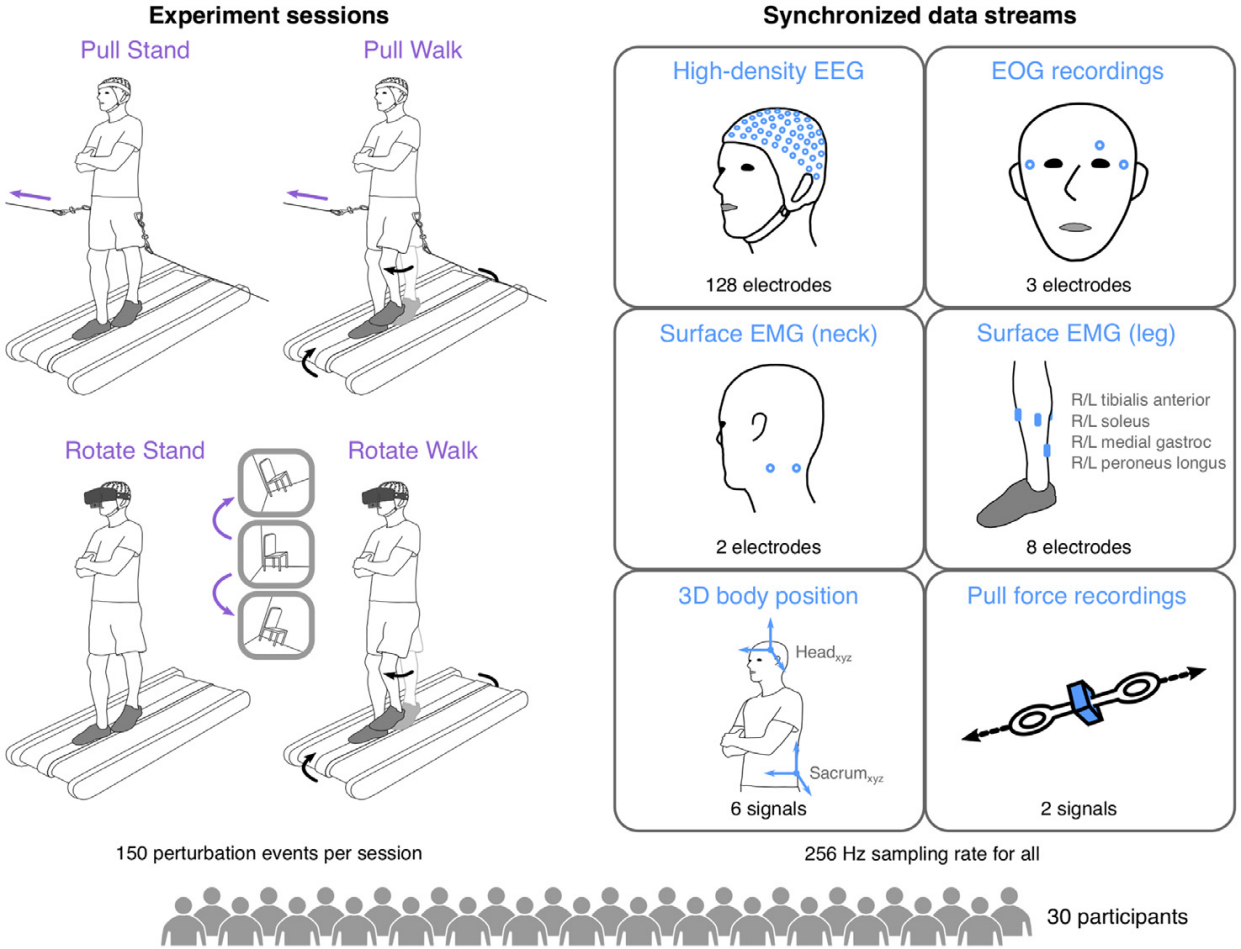
*Experiment design and overview of recorded data streams.* Our dataset was recorded from 30 participants during four 10 min sessions where participants were exposed to brief side-to-side pulls or visual field rotations while either walking or standing on a treadmill-mounted balance beam. Each session contains 150 perturbation events (75 in each direction). During each session, high-density electroencephalography (EEG), electrooculography (EOG), surface electromyography (EMG), three-dimensional body position (via motion capture), and pull force were recorded and synchronized at a 256 Hz sampling rate.

In each session, participants were exposed to one of two sensorimotor perturbations: a virtual-reality-induced visual field rotation or a mediolateral pull at the waist (Fig. 1). We used an Oculus Rift DK2 virtual reality headset to present visual field rotation perturbations by displaying a passthrough view from a video camera mounted to the headset (Logitech C930e; Logitech, Lausanne, Switzerland), located near the participant’s nose. At the onset of each rotation perturbation, this passthrough view was instantly rotated 20° clockwise or counterclockwise using Unity 5 software (Unity Technologies, San Francisco, USA). This rotated view lasted for 0.5 s before instantaneously reverting to the original, unrotated view. We performed mediolateral pull perturbations using two electromechanical motors placed on either side of each participant. Each motor was fastened to one end of a thin 30.5 cm-long metal bar, with a steel cable connected at the other end. This cable was attached to the body-support harness close to the participant’s waist. At the start of each pull perturbation, one motor would be commanded (dSPACE GmbH, Paderborn, Germany) to rotate the attached bar 90° away from the participant for 1 s, pulling the participant towards their left or right.

Participants were separately exposed to each perturbation type while either walking or standing, resulting in four sessions total. During each session, participants were perturbed 150 times (75 in each direction) in a pseudo-random sequence. For each perturbation type, participants always performed the standing session first, followed by the walking session. We randomly selected half of the participants to perform the rotation sessions first while the other half was exposed to the pull perturbations first.

### 2.2 Data Acquisition

We recorded multiple biosignals during each session, including high-density EEG, EOG, neck and lower leg EMG, and motion capture (Fig. 1). We performed EEG recordings with a 136-electrode BioSemi ActiveTwo system with gelled electrodes (512 Hz sampling rate; BioSemi BV, Amsterdam, Netherlands). All electrode positions were precisely measured using an ELPOS Digitizer (Zebris Medical GmbH, Isny, Germany). We used two of the BioSemi electrodes to measure posterior neck muscle activity. In addition, we placed three BioSemi electrodes around the eyes to record EOG (see Fig. 1 for placement). We recorded surface EMG (1000 Hz sampling rate; Biometrics. Ltd, Newport, UK) from 4 lower leg muscles on each leg: tibialis anterior, soleus, medial gastrocnemius, and peroneus longus. We selected leg muscles that were relevant to walking and mediolateral balance. In addition, we recorded three-dimensional positions of the head and sacrum using reflective motion capture markers, sampled at 100 Hz. We also attached tensile load cells (1000 Hz sampling rate; Omega Engineering, Norwalk, USA) in series with the both cables to record pull perturbation force and onset times. Leg EMG, motion capture, and load cell data streams were recorded synchronously using Vicon Nexus software (Vicon Motion Systems, Oxford, UK).

### 2.3 EEG, EOG, and neck EMG pre-processing

We pre-processed EEG, EOG, and neck EMG together using custom EEGLAB scripts [6]. Data were downsampled to 256 Hz, high-pass filtered at 1 Hz, referenced to the common median of all electrodes, and processed with Cleanline to minimize line noise at 60 Hz and its harmonics (https://github.com/sccn/cleanline). We also identified noisy EEG electrodes that had abnormally high standard deviation, had kurtosis >5 standard deviations above the average electrode, or were uncorrelated for >1% of the time [12], [13]. This process identified 17 ± 7 (mean ± SD) noisy electrodes per participant.

### 2.4 Data synchronization and alignment

We synchronized all data streams using a 0.5 Hz square pulse sent to every recording device. All data streams were aligned to the pre-processed EEG data with 256 Hz sampling. Prior to alignment, we low-pass filtered the leg EMG and load cell data using a 4th order Butterworth filter with a 250 Hz cutoff frequency to avoid anti-aliasing effects. We also identified and visually verified corresponding sync rising and falling edges across data streams. Next, we used the timing of each rising and falling edge in order to segment the leg EMG, motion capture, and load cell data streams such that each ∼1 s segment started and ended when the sync signal either rose or fell. Because the sync rising and falling edges are aligned across all signals, these segments are synchronized across data streams, but need to be resampled to match the EEG sampling rate. To achieve this, we interpolated each segment to the number of EEG timepoints between the corresponding sync signal edges using MATLABs *interp1* function. We chose this interpolation procedure to minimize alignment errors due to dropped frames in any of the data streams.

### 2.5 Perturbation Event Timings

We identified the onset times for both sensorimotor perturbation types. For visual field rotations, we programmed virtual keyboard button presses to occur at the onset of each rotation, with different keys distinguishing between clockwise and counterclockwise rotations. These button presses were automatically synchronized to the EEG recordings using Lab Streaming Layer [14]. We estimated pull perturbation onset times by identifying the peaks in detrended load cell data and then finding when the load cell first went 3 standard deviations above baseline voltage before each peak. We visually inspected all peak detections and pull onset event times to ensure accuracy.

## Ethics Statement

All research was carried out in accordance with The Code of Ethics of the World Medical Association (Declaration of Helsinki). Our protocol was approved by the University of Michigan Health Sciences and Behavioral Sciences Institutional Review Board (protocol number: HUM00117824) for the protection of human participants. All participants provided written informed consent before participating.

## CRediT authorship contribution statement

**Steven M. Peterson:** Conceptualization, Methodology, Software, Validation, Data curation, Visualization, Writing – original draft, Writing – review & editing. **Daniel P. Ferris:** Conceptualization, Writing – review & editing.

## Declaration of Competing Interest

The authors declare that they have no known competing financial interests or personal relationships which have, or could be perceived to have, influenced the work reported in this article.
